# Proton dose distribution measurements using a MOSFET detector with a simple dose‐weighted correction method for LET effects

**DOI:** 10.1120/jacmp.v12i2.3431

**Published:** 2011-04-04

**Authors:** Ryosuke Kohno, Kenji Hotta, Taeko Matsuura, Kana Matsubara, Shie Nishioka, Teiji Nishio, Mitsuhiko Kawashima, Takashi Ogino

**Affiliations:** ^1^ National Cancer Center Hospital East Chiba 277‐8577 Japan; ^2^ National Cancer Center Research Institute Tokyo 104‐0045 Japan; ^3^ Pure and Applied Sciences University of Tsukuba Tsukuba Ibaraki 305‐8577 Japan; ^4^ Research Fellow of the Japan Society for the Promotion of Science; ^5^ Department of Medical Physics Hokkaido University Hospital Hokkaido 060‐8648 Japan; ^6^ Graduate School of Human Health Sciences Tokyo Metropolitan University Tokyo 116‐8551 Japan; ^7^ Foundation for Promotion of Cancer Research Tokyo 104‐0045 Japan

**Keywords:** proton, MOSFET detector, LET, simple dose‐weighted correction method, *in vivo* dosimetry

## Abstract

We experimentally evaluated the proton beam dose reproducibility, sensitivity, angular dependence and depth‐dose relationships for a new Metal Oxide Semiconductor Field Effect Transistor (MOSFET) detector. The detector was fabricated with a thinner oxide layer and was operated at high‐bias voltages. In order to accurately measure dose distributions, we developed a practical method for correcting the MOSFET response to proton beams. The detector was tested by examining lateral dose profiles formed by protons passing through an L‐shaped bolus. The dose reproducibility, angular dependence and depth‐dose response were evaluated using a 190 MeV proton beam. Depth‐output curves produced using the MOSFET detectors were compared with results obtained using an ionization chamber (IC). Since accurate measurements of proton dose distribution require correction for LET effects, we developed a simple dose‐weighted correction method. The correction factors were determined as a function of proton penetration depth, or residual range. The residual proton range at each measurement point was calculated using the pencil beam algorithm. Lateral measurements in a phantom were obtained for pristine and SOBP beams. The reproducibility of the MOSFET detector was within 2%, and the angular dependence was less than 9%. The detector exhibited a good response at the Bragg peak (0.74 relative to the IC detector). For dose distributions resulting from protons passing through an L‐shaped bolus, the corrected MOSFET dose agreed well with the IC results. Absolute proton dosimetry can be performed using MOSFET detectors to a precision of about 3% (1 sigma). A thinner oxide layer thickness improved the LET in proton dosimetry. By employing correction methods for LET dependence, it is possible to measure absolute proton dose using MOSFET detectors.

PACS number: 87.56.‐v

## I. INTRODUCTION

The Metal Oxide Semiconductor Field Effect Transistor (MOSFET) detector is widely used as a pinpoint dosimeter for photon and electron dose verification.^(^ ^1^ ^–^ ^6^ ^)^ The typical design uses a p‐channel enhanced MOSFET constructed on a negatively doped (n‐type) silicon substrate. Ionizing radiation generates electron‐hole pairs in the insulating layer. The holes drift toward the substrate under an appropriate bias voltage and are semipermanently trapped at the interface, resulting in a shift in the gate voltage required for source‐drain conductivity that is proportional to the radiation dose. Following exposure, the gate threshold voltage is measured by applying a constant source‐drain current, and the cumulative dose is obtained using suitable calibration factors. The major advantages of this detector include small physical size, the ability to permanently store the accumulated dose, dose‐rate and temperature independence, real‐time readout, roughly isotropic response for photon beams, and ease of use.

Kohno et al.^(7)^ evaluated the use of the commercially available TN‐502RD MOSFET detector with oxide thicknesses of 0.5 μm (Best Medical Canada, Ottawa, Canada) for proton dose measurement. The dose reproducibility, linearity, fading effect and beam intensity dependence were similar to the response obtained from photon beams. On the other hand, Bragg curves measured using the TN‐502RD at high bias settings were 20%–40% lower than those measured using an ionization chamber. The MOSFET response is strongly dependent on the degree of linear energy transfer (LET) occurring through columnar recombination. This is due to the significant reduction in charge recombination when the electric field applied to the MOSFET is perpendicular to the plasma track, leading to faster drift of electron‐hole pairs. As a result of the LET dependence and the columnar recombination effect, quantitative proton dose measurements are difficult to accurately perform using MOSFET detectors. In order to use a MOSFET detector for proton dosimetry, improved characterization of the response in the Bragg peak region is necessary. Kohno et al.^(7)^ also reported that the response of the TN‐502RD was approximately 15% higher than the IC detector at most angles. A lower angular dependence would be desirable when using MOSFET detectors for *in vivo* proton dosimetry.

Cheng et al.^(8)^ investigated another OneDose single use MOSFET detector (Sicel Technologies, Inc., Morrisville, NC) for *in vivo* dosimetry in proton beam therapy. The OneDose detector generally underresponsed compared to the Markus chamber, about 5% at depth of ~ 5cm, and increase to ≤ 200% at the Bragg peak and beyond. Although it is difficult to measure the Bragg peak with the OneDose, the Cheng study reported that the OneDose provides an opportunity to measure surface dose with proton beam within acceptable clinical criterion of ± 5.0%−6.5%.

In this study, we examined a new MOSFET detector with an oxide thickness of 0.25 μm (TN‐252RD) to improve characterization of the MOSFET response for proton beams. The dose sensitivity, angular dependence, and depth‐dose response were experimentally evaluated at high bias settings using a 190 MeV proton beam. We also implemented a simple dose‐weighted correction method to account for LET dependence suitable for clinical applications. This method was used to perform absolute proton dosimetry using the MOSFET detector.

## II. MATERIALS AND METHODS

### A. MOSFET dosimetry system

A commercially available MOSFET patient dose verification system (Best Medical Canada, Ottawa, Canada) was used. In order to reduce temperature dependence and nonlinear response at high‐dose levels,^(9)^ the dual‐MOSFET is composed of two identical MOSFETs, fabricated on the same silicon substrate, with an active area of $0.2\times 0.2\;{\text{mm}}^{2}$. This placement allows for temperature compensation as the two MOSFETs are located on the same substrate. The oxide thickness for the TN‐252RD MOSFET is 0.25 μm. The detectors are 2×1.3×8 mm in size including the encapsulation.^(10)^ All measurements were performed using a high‐sensitivity bias voltage setting.

### B. Experimental apparatus

#### B.1 Proton beam setup

Measurements were carried out using the therapeutic proton beam line at the National Cancer Center Hospital East, Japan. The beam line employs the dual‐ring double‐scattering method for proton therapy.^(11)^ The thickness of the first scatter and the shape of the second scatter are determined by the energy of the proton beams. The maximum size of the irradiation field provided by this system is 200 mm in diameter. The energy of the proton beam was maintained at 190 MeV, and daily testing was used to ensure the proton range was within ± 0.5 mm.^(12)^

#### B.2 MOSFET sensitivity and dose calibration

In MOSFET sensitivity (mV/cGy) measurements, the proton energy was 157 MeV at a detector located within a PMMA dose calibration phantom. At this energy the MOSFET detectors displayed no response changes due to LET dependence. A calibrated 0.6 cc Farmer ionization chamber (IC) type 30013 (PTW, Freiburg, Germany) and MOSFET detector were placed along a line perpendicular to the beam axis. The MOSFET and the IC were exposed five times to 200 cGy, and the MOSFET sensitivity was determined from the average output. The sensitivity of the MOSFET detector was also measured using proton beams with energies of 50, 100, 150, 157 and 200 MeV.

For accurate comparisons, the detector outputs were converted to dose values. The dose calibration factor $\left(F_{\text{calib}}\right)$ in cGy/mV for the MOSFET detector was measured using a 157 MeV proton beam. The raw dose $\left(D_{\text{raw}}\right)$ for the MOSFET detector was obtained from the product of the MOSFET reading *R* in mV and the dose calibration factor:

(1) $$D_{raw}=F_{calib}\times R$$

#### B.3 Angular dependence

The response of MOSFET detectors is dependent on the angle of incidence.^(^ ^1^ ^–^ ^5^ ^,^ ^7^ ^)^ The angular dependence was experimentally evaluated using a cylindrical acrylic phantom with a radius of 8 cm and a length of 15 cm. The angular response with respect to the cable axis was measured at 30°, 45°, 60°, 90°, 120°, 135°, 150°, and 180°.

#### B.4 Depth‐dose curves

Depth‐dose curves for mono‐energetic proton beams were determined using the IC and MOSFET detectors. Polyethylene (PE) slabs ranging in thickness from 0 to 175 mm were stacked on top of the calibration phantom containing the detectors. The equivalent water thickness was calculated by multiplying the polyethylene thickness by 1.02. The measurements were repeated three times at each thickness, and the results were normalized with respect to the response at a thickness of 0 mm. The ratio of the response of the IC detector to the MOSFET detector was also plotted as a function of thickness. The correction factors (IC/MOSFET) were expressed as a function of the PE thickness $(d_{\text{PE}}):{\text{cf}}_{\text{mono}}$ ($d_{\text{PE}}$).

In actual proton therapy, most patients are treated using a SOBP proton beam created using a ridge‐filter. We therefore also measured the depth‐dose distribution of an 80 mm SOBP‐width proton beam using the MOSFET detector. The ratio of the IC response to the MOSFET response (IC/MOSFET) was obtained and the correction factor ${\text{cf}}_{\text{SOBP}}$ was determined as a function of $d_{\text{PE}}$ as was done for the mono‐energetic proton beam.

### C. Dose distribution formed by the protons traversing an L‐shaped bolus

#### C.1 Experimental apparatus

**Figure 1:**
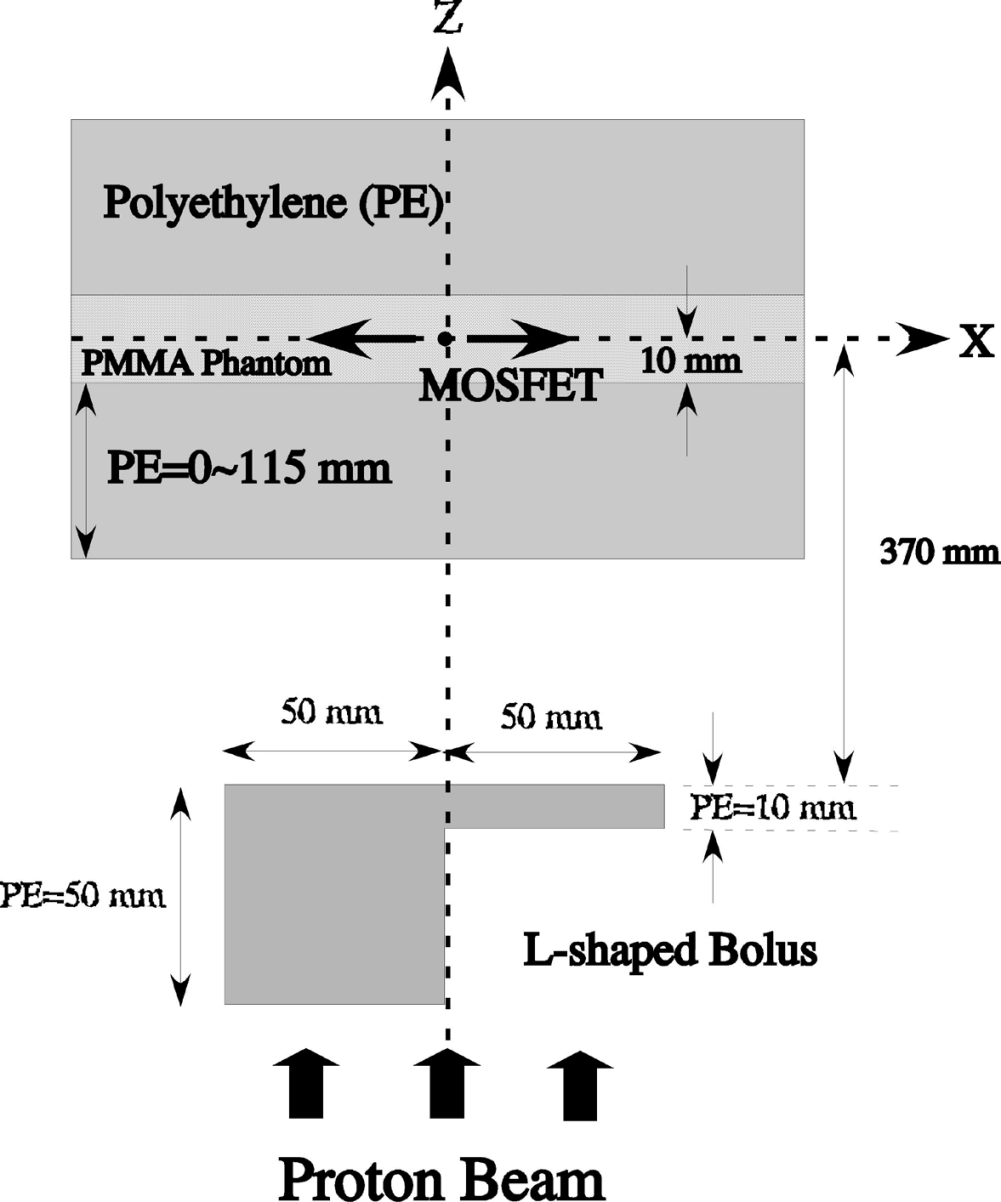
Experimental arrangement for measurement of dose distribution (top view). The bolus was made of polyethylene.

We prepared a polyethylene bolus with an L‐shaped horizontal cross section (Fig. 1). The bolus was 50 mm thick at points where x<0 and 10 mm thick when x≥0. This bolus shape was selected to correspond to the target with large heterogeneity in the lateral direction. Particularly, we expect lateral dose distributions around x=0 form a complex bump and dip structure due to the bolus edge scattering effect of the bolus region where the thickness changes abruptly. The correction factor of the MOSFET response must take these effects into consideration.

For the 190 MeV mono‐energetic proton beam, the Bragg peak positions were 110 mm for protons passing through the thicker section and 150 mm for protons passing through the thinner section. Polyethylene slabs of various thicknesses were stacked on top of the PMMA calibration phantom. The lateral (x‐axis) dose distributions were measured using the IC and the TN‐252RD MOSFET detector at PE thicknesses of 0, 100, 105, 110 and 115 mm. In addition, we measured the lateral dose distributions at PE thicknesses of 0, 50 and 100 mm for the 80 mm SOBP‐width beam.

#### C.2 A simple dose‐weighted correction method

Because of LET dependence, there is a notable disagreement between the IC and the TN‐502RD MOSFET detector near the Bragg peak.^(7)^ Knowledge of the LET spectrum is important in cavity theory to account for recombination effects and stopping power ratios.^(13)^ The difference in response cannot be completely explained by differences in stopping power between water and ${\text{SiO}}_{2}$. In addition, proton beam therapy uses a spread‐out Bragg peak (SOBP) beam containing protons with a range of energies, making it difficult to easily and accurately calculate the LET spectrum at a particular measurement point due to bolus and tissue heterogeneities.

In order to provide a simple correction for the response of the MOSFET detector to various LET effects, we employed a method originally used to correct imaging plate response.^(14)^ A Bragg curve was obtained using the IC detector to establish a standard for the proton beam depth‐dose distribution. This curve was then used to calculate correction factors (IC/MOSFET) as a function of proton penetration depth.

The proton penetration depth can be considered as a residual range. Since the protons at any point have a variety of energies due to multiple scattering effects, the residual proton range at an arbitrary point may be calculated using the pencil beam dose calculation algorithm (PBA),^(^ ^15^ ^–^ ^17^ ^)^ in which the pencil beam dose distribution is separated into a central‐axis term and an off‐axis term. The central‐axis term represents the measured depth‐dose distribution of the broad beam. The off‐axis term is a two‐dimensional Gaussian distribution the standard deviation of which corresponds to the lateral beam spread. The dose $F(x,y,z,(x_{0},y_{0}))$ delivered by a single pencil beam at an entrance position $\left(x_{0},y_{0}\right)$ is given by:

(2) $$\begin{array}{l} F(x,y,z);(x_{0},y_{0})=\phi (x_{0},y_{0})DD(z;(x_{0},y_{0}))\\ \;\times \frac{1}{2\pi \sigma {\left(z\right)}^{2}}\exp\left(-\frac{{\left(x_{0}-x\right)}^{2}-{\left(y_{0}-y\right)}^{2}}{2\sigma {\left(z\right)}^{2}}\right), \end{array}$$

where $\phi (x_{0},y_{0})$ is the intensity profile of the broad beam, $\text{DD}(z;(x_{0},y_{0}))$ is the depth‐dose distribution of the broad beam, and σ(*z*) is the proton spread due to multiple scattering effects in the bolus and polyethylene slabs and the configuration of the beam line at *z*. We can obtain the dose distribution in the region of interest by generating many pencil beams and summing their dose distributions. For dose distributions of protons traversing an L‐shaped phantom, Kohno et al.^(16)^ reported the precision of doses calculated using the PBA is approximately 2.5%. The PBA may therefore be considered a precise and practical method for calculating the proton residual range in order to obtain correction factors at arbitrary locations.

The correction factor for the MOSFET response CF(x,y,z) is given by:

(3) $$CF(x,y,z)=\frac{\sum_{i=1}^{n}cf_{dd}(z;(x_{i},y_{i}))F(x,y,z;(x_{i},y_{i}))}{\sum_{i=1}^{n}F(x,y,z;(x_{i},y_{i}))}$$

in which *i* is the *i*th pencil beam, *n* is the total number of pencil beams, $(x_{i},y_{i})$ is the position of a generated pencil beam, and $cf_{dd}(z;(x_{i},y_{i}))$ is $cf_{mono}(z-d_{PE})$ or $cf_{SOBP}(z=d_{PE})$ (as described in Section B.4 above). The dose measured by the MOSFET detector at (x,y,z), D(x,y,z) may be calculated using:

(4) $$D(x,y,z)=CF(x,y,z)\cdot D_{raw}(x,y,z),$$

where $D_{\text{raw}}(x,y,x)$ is the raw dose (as described in Section B.2 above).

Proton dose distributions resulting from an L‐shaped bolus (Fig. 1) were measured using the MOSFET and the IC detectors. Protons passing near the abrupt change in thickness at x=0 displayed a range of energies due to multiple scattering effects, and it was necessary to calculate the proton residual range using the PBA in order to obtain the correction factor.

## III. RESULTS & DISCUSSION

### A. Dose sensitivity

**Figure 2:**
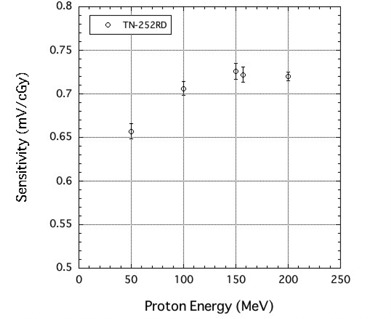
MOSFET sensitivity for 200, 157, 150, 100 and 50 MeV proton beams.

The sensitivity of the TN‐252RD MOSFET detector was 0.72±0.01 (mV/cGy) and the corresponding reproducibility was ± 1.4%. Although the sensitivity of this detector was lower than the TN‐502RD MOSFET with a thicker oxide layer, its reproducibility was within 2%. Figure 2 is a graph of the TN‐252RD MOSFET sensitivity for each proton energy value. The sensitivities to 150, 157, and 200 MeV proton beams were almost identical, but the sensitivity was reduced at lower proton energies of 100 and 50 MeV.

### B. Angular dependence

**Figure 3:**
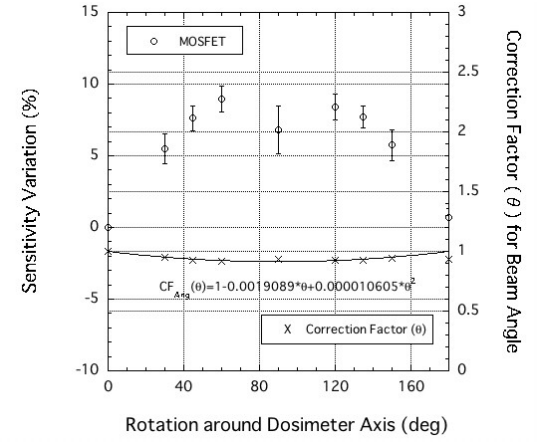
Angular dependence of MOSFET detectors exposed to a 190 MeV proton beam. The correction value for the angular response of the MOSFET detector is also plotted.

Figure 3 depicts an angular dependence of the MOSFET detector exposed to a 190 MeV proton beam, and the correction value for the angular response of the MOSFET detector. The electric field is parallel to the incident proton beam when the MOSFET detector is mounted at 0 degrees. The response was normalized to 0°, corresponding to a beam perpendicular to the MOSFET encapsulation epoxy. The angular response at 180° agreed well with the 0° measurements (within ± 2.0%). The TN‐252RD detector displayed a maximum overresponse of +9.0%. The overresponse occurs because the fraction of charge pairs escaping recombination increases at larger angles between the electric field and the proton track.^(18)^ Despite the large value, this is a dramatic improvement of almost 10% relative to the TN‐502RD device,^(7)^ suggesting that MOSFET detectors constructed using thinner ${\text{SiO}}_{2}$ layers exhibit reduced angular dependence. The correction value ${\text{CV}}_{\text{Ang}}(\theta )$ may be obtained from the angular response of the TN‐252RD detector at a beam angle θ using the relation:

(5) $$CV_{Ang}(\theta )=1-0.00197\cdot \theta +0.0000109\cdot \theta^{2}.$$

Using this correction value, we can correct the angular response of the TN‐252RD MOSFET detector to within 1.5%.

### C. Depth‐dose curves

**Figure 4:**
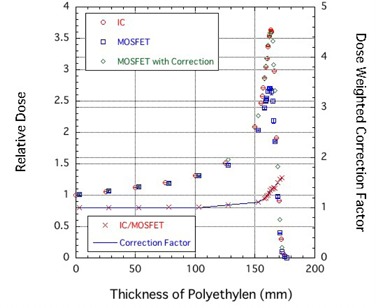
Comparison of Bragg curves obtained using IC and MOSFET detectors at high‐bias setting for a 190 MeV proton beam. The correction factor for the response of the MOSFET detector was calculated as a function of proton penetration depth.

Figure 4 shows a comparison of Bragg curves obtained using IC and MOSFET detectors at high‐bias setting for a 190 MeV proton beam, and the correction factor for the response of the MOSFET detector was calculated as a function of proton penetration depth. The relative response of the TN‐252RD MOSFET detector at the Bragg peak was 0.74. This response relative to the TN‐502RD detector^(7)^ also is a larger than a 10% improvement. The correction factor $cf_{\text{mono}}(z=d_{PE})$ for the response of the MOSFET detector was determined as a function of proton penetration depth as follows:

(6) $$cf_{mono}(d_{PE})=\{\begin{array}{ll} \;1 & \left[d_{PE}<100.421\;(mm)\right]\\ \;0.781885+0.002172\cdot d_{PE} & \left[100.421\;(mm)\leq d_{PE}<154.784\;(mm)\right]\\ -2.94139+0.0262266\cdot d_{PE} & \left[154.784\;(mm)\leq d_{PE}\right] \end{array}.$$

The MOSFET with the correction agreed well with the IC within 1.5%, as shown in Fig. 4.

**Figure 5:**
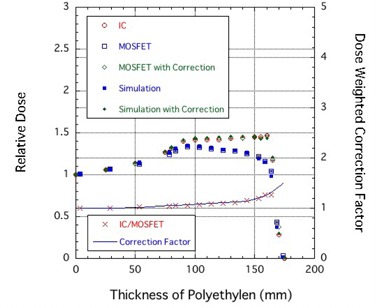
Comparison of SOBP obtained using IC and MOSFET detectors. The correction factor for the response of the MOSFET detector was calculated as a function of proton penetration depth.

A comparison of the SOBP obtained using the IC and MOSFET detectors is shown in Fig. 5. Figure 5 also shows the correction factor for the response of the MOSFET detector was calculated as a function of proton penetration depth. The ratio of the IC and MOSFET (IC/MOSFET) response was also obtained. The correction factor ${\text{cf}}_{\text{SOBP}}(d_{\text{PE}})$ was expressed as a function of PE thickness using:

(7) $$cf_{SOBP}(d_{PE})=\{\begin{array}{ll} \;1 & \left[d_{PE}<40\;(mm)\right]\\ 0.94639+0.00134025\cdot d_{PE} & \left[40\;(mm)\leq d_{PE}<140\;(mm)\right]\\ 6.59257-0.0793194\cdot d_{PE}+0.00028807\cdot d_{PE}^{\;\;\;\;\;\;2} & \left[140\;(mm)\leq d_{PE}\right] \end{array}$$

The MOSFET with the correction agreed well with the IC within 1.4%, as shown in Fig. 5.

In this method, ${\text{cf}}_{\text{SOBP}}(d_{\text{PE}})$ must be measured and calculated for each SOBP width. However, a SOBP distribution may be obtained in a stepwise manner from the dose contributions of mono‐energetic proton beams traversing the individual elements of the ridge filter. For example, the “Simulation” curve in Fig. 5 depicts the SOBP distribution obtained using the uncorrected depth‐output curve measured with the MOSFET detector. The “Simulation with Correction” curve depicts results corrected without the necessity of applying the experimentally determined MOSFET response corrections to the “Simulation” curve. Thus, given ${\text{cf}}_{\text{mono}}(d_{\text{PE}})$ of Eq. (6) for the mono‐energetic proton beam, we can obtain ${\text{cf}}_{\text{SOBP}}(d_{\text{PE}})$ for various SOBP‐width proton beams by simulating the SOBP beam using the Bragg curve of a mono‐energetic proton beam.

### D. Bolus experiments

**Figure 6:**
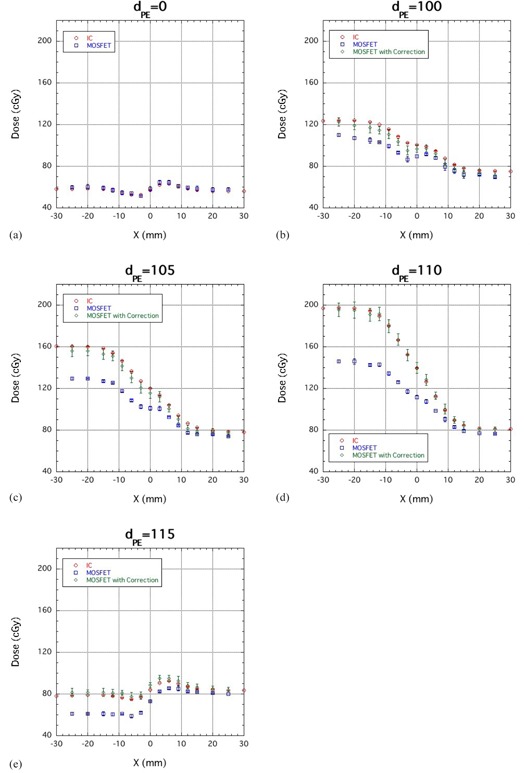
Comparison of lateral‐dose distribution obtained using IC, uncorrected MOSFET (MOSFET) and corrected MOSFET detectors (MOSFET with Correction) at PE thicknesses of 0 (a), 100 (b), 105 (c), 110 (d) and 115 (e) mm for a 190 MeV mono‐energetic proton beam.

Figure 6 compares the lateral dose distributions obtained for a 190 MeV proton beam using IC and MOSFET detectors at PE thicknesses of 0 (a), 100 (b), 105 (c), 110 (d) and 115 (e) mm. The error bar in Fig. 6 includes the reproducibility of the MOSFET measurements and calculation errors of 3% to account for uncertainties in the PBA (as described in Materials and Methods Section C.2). In Fig. 6(a), a bump and dip structure is evident near x=0. This is the result of edge scattering effects due to the abrupt change in thickness. The uncorrected MOSFET results agreed well with the IC measurements, and the MOSFET response due to LET did not change at this depth. Thus, in shallow regions, depth‐dose distribution corrections are unnecessary.

On the other hand, the MOSFET detector response began to change at x<0 and the uncorrected MOSFET output deviated significantly from the IC response (Fig. 6(b). Because the depth at x<0 is close to the Bragg peak position, the MOSFET response was reduced. Since edge scattering causes the lateral dose distribution near x=0 to be determined by protons with a distribution of energies, we expected that changes in the MOSFET response would be complex. However, the corrected output of the MOSFET detector agreed well with the IC results within an average difference of 4.4%, demonstrating that MOSFET detectors are suitable for proton dosimetry when the response is corrected. Despite the drastic change in MOSFET detector response near x<0 for PE thicknesses of 105, 110 and 115 mm, the corrected output agreed with the IC results ((Figs. 6c), 6(d), and 6(e)) within 3.2% (1 sigma).

**Figure 7:**
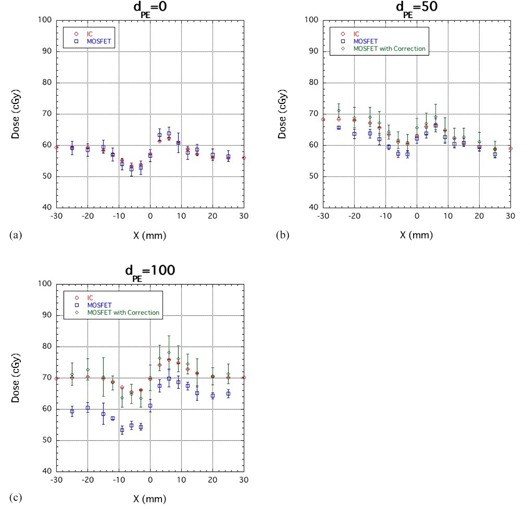
Comparison of lateral‐dose distribution measurements obtained using IC, uncorrected MOSFET (MOSFET) and corrected MOSFET detectors (MOSFET with Correction) at PE thicknesses of 0 (a), 50 (b) and 100 (c) mm for a SOBP proton beam.

Figure 7 is a comparison of the lateral‐dose distribution obtained using the IC and MOSFET detectors at PE thicknesses of 0 (a), 50 (b) and 100 (c) mm for an SOBP proton beam. The corrected MOSFET output agreed well with the IC results. For the SOBP beam, the accuracy of the dose measurement was approximately 2.3% (1 sigma).

By employing correction methods for LET and angular dependence, it is possible to perform *in vivo* proton dosimetry using a MOSFET detector. However, the correction method for LET effects is highly dependent on the precision of the PBA calculation, and further improvements to the dose calculation algorithm (for instance the application of Monte Carlo methods) would be desirable in situations involving tissues with significant heterogeneity.^(^ ^19^ ^–^ ^23^ ^)^

## IV. CONCLUSIONS

We experimentally evaluated the proton beam dose reproducibility, angular dependence and depth‐dose relationships for a new TN‐252RD MOSFET detector at high‐bias voltages. The reproducibility of the MOSFET detector was within 2%, and the angular dependence was less than 9%. For depth‐dose distribution measurements, the relative response of the MOSFET detector at the Bragg peak region was 26% lower than measurements obtained using an ionization chamber. A thinner oxide layer thickness improved the LET dependence in proton dosimetry, although LET dependence was still the limiting factor in accurate depth‐dose estimation.

In order to measure dose distributions using a MOSFET detector, we developed a practical method for correcting the MOSFET response to proton beams. For dose distributions resulting from protons passing through an L‐shaped bolus, the corrected MOSFET dose agreed well with the IC results. Absolute proton dosimetry was performed using MOSFET detectors with a precision of approximately 3% (1 sigma), and from this we conclude that it is possible to measure proton doses using MOSFET detectors.

## ACKNOWLEDGMENTS

We thank Dr. A. Hallil, Best Medical Canada, for his support in the form of materials and special prototypes. We are grateful to Kazutomo Matsumura, Hideki Saitoh, Toshinobu Sasano, Naoya Uzawa and Ryuichi Oota, SHI Accelerator Service Ltd, for experimental support. The authors wish to thank Akihiro Nohtomi, Ph.D. for a critical review of the manuscript. This work was supported in part by a Grant‐in‐Aid for Young Scientists (B) No. 21791236 from the Japan Society for Promotion of Science (JSPS).

## Supporting information

Supplementary Material Files
