# Supplementary figures and images for: Proton dose distribution measurements using a MOSFET detector with a simple dose‐weighted correction method for LET effects
Source: J Appl Clin Med Phys. 2011 Apr 4;12(2):326–37. doi: 10.1120/jacmp.v12i2.3431 (PMC5718688; doi:10.1120/jacmp.v12i2.3431)

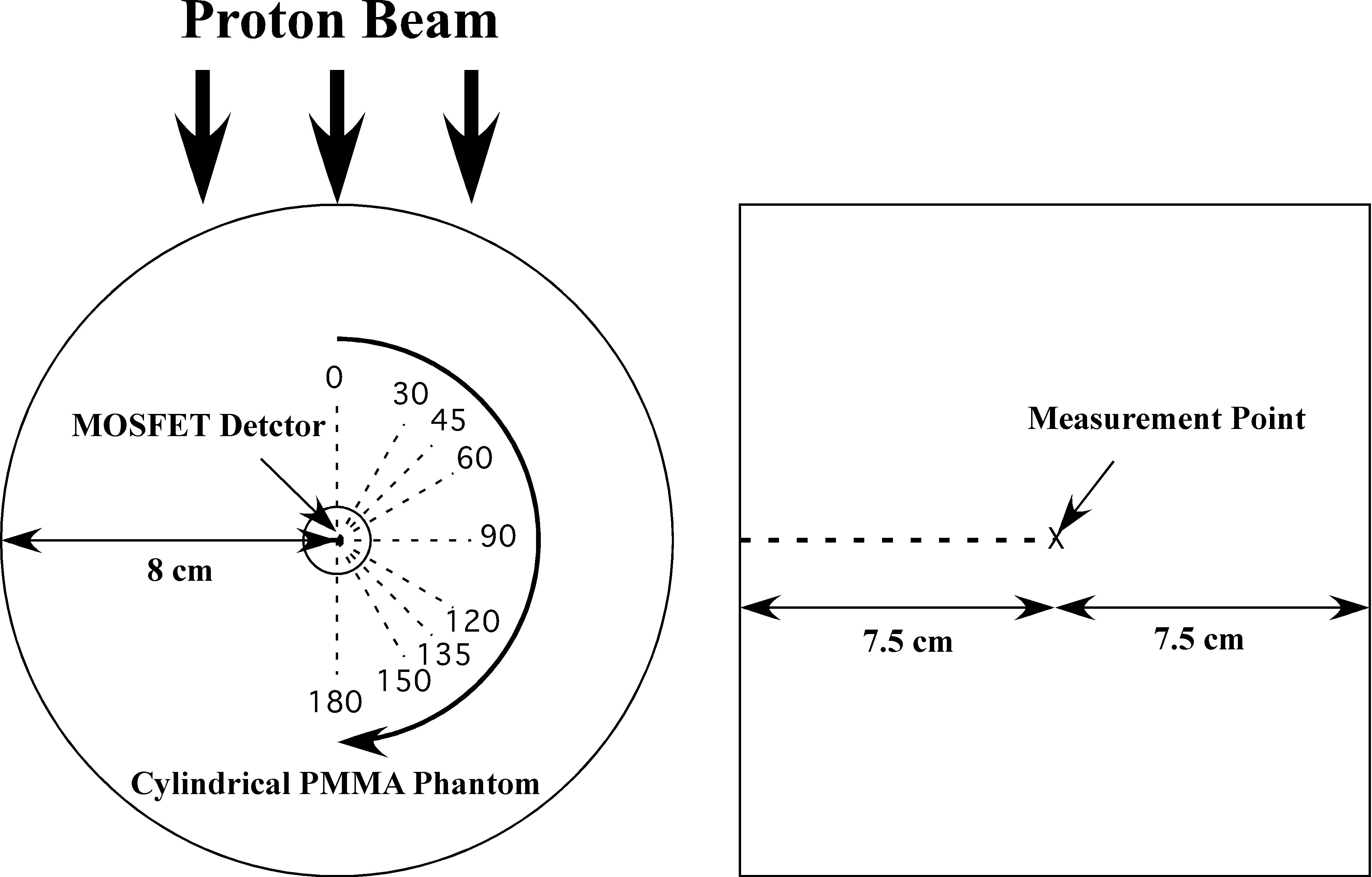

Supplement: Supplementary file 1 — Supplementary Material Files [file ACM2-12-326-s001.jpg]
